# Thermo‐tone: A newly revealed mechanism of blood flow regulation

**DOI:** 10.1113/EP093562

**Published:** 2026-10-15

**Authors:** Thieu X. Phan, Gerard P. Ahern

**Affiliations:** ^1^ Department of Pharmacology and Physiology Georgetown University Washington DC USA; ^2^ Department of Biology Vinh University Vinh City Vietnam

**Keywords:** myogenic tone, temperature, TRP channels

## Abstract

Myogenic tone enables dynamic control of tissue blood flow. This intrinsic property of vascular smooth muscle cells to sense stretch and reflexively adjust the calibre of small arteries is crucial to maintain capillary pressure and a blood flow reserve. For over 120 years blood pressure was considered the sole determinant of myogenic tone. Our recent research reveals an equal, critical role for temperature. This property, termed ‘thermo‐tone’, renders the tone of arteries strictly heat dependent with thermosensitivities tuned close to resting tissue temperatures (25–37°C). Here, we discuss the underlying signalling mechanisms and the potential role for thermo‐tone in the homeostatic regulation of blood flow.

## Abstract (highlights)

- **What is the topic of this review?**
  Temperature is well known to affect blood flow, which is largely attributed to autonomic neurogenic mechanisms. This review discusses the recent discovery that arteries are intrinsically temperature‐sensitive and generate myogenic tone by integrating both pressure and temperature signals.
- **What advances does it highlight?**
  Recent findings highlight an important role for local tissue temperature in the homeostatic regulation of blood perfusion.

## 1 VASCULAR MYOGENIC TONE

The cardiovascular system delivers blood to every organ, transporting O_2_/CO_2_, nutrients and also regulating body temperature. To achieve these goals, cardiac output and tissue blood flow must be tightly controlled and in a tissue‐specific manner. Arteries are lined with smooth muscle cells that contract or relax to adjust the vessel diameter. In turn, various extrinsic signals, neurotransmitters (noradrenaline), hormones (like adrenaline) and endothelial‐derived mediators (nitric oxide) regulate vascular smooth muscle contractility. In addition to these extrinsic modulators, vascular smooth muscle cells intrinsically respond to changes in local blood pressure via a mechanism called myogenic tone. Myogenic tone is a fundamental property of arterial smooth muscle cells, directing vessels to constrict in response to an increase in pressure and dilate in response to a decrease in pressure and thus maintain a constant blood flow (Davis, 2012; Davis et al., 2023). Although the precise physiological roles are still debated, at least two important haemodynamic functions are attributed to myogenic tone. First, myogenic tone serves to stabilize tissue blood flow over a range of blood pressures (∼40–150 mmHg), thus augmenting flow to under‐perfused tissues and protecting downstream capillaries from damaging high pressure. Second, myogenic tone establishes a blood flow reserve. A fully pressurized artery will have upwards of 50% myogenic tone. This resting tone enables vasodilator signals to locally increase tissue blood flow. Notably, myogenic tone is impaired in diseases states including ageing (Ghosh et al., 2015; Lott et al., 2004), diabetes and cardiovascular disease (Duncker et al., 2015; Hodnett & Hester, 2007; Petersen et al., 2002) emphasizing an important homeostatic role for this haemodynamic mechanism.

Although Sir William Bayliss first described myogenic tone more than 120 years ago (Bayliss, 1902), details of the underlying mechanisms are still emerging. Although originally described in vivo, the gold standard technique for studying myogenic tone is pressure myography in which isolated arteries are cannulated and mechanically pressurized to mimic in vivo conditions. Increasing the intraluminal pressure produces a circumferential stretch of the vascular smooth muscle, which in turn triggers membrane depolarization and opening of the voltage‐gated Ca^2+^ channel Ca_V_1.2 (Earley & Brayden, 2015; Knot & Nelson, 1998). The resulting increase in myoplasmic Ca^2+^ triggers muscle contraction. In addition, mechano‐activated Rho A–Rho kinase signalling contributes to the Ca^2+^ sensitivity of the smooth muscle allowing for sustained contraction (Gokina et al., 2005).

How is intraluminal pressure sensed? While, mechano‐activated ion channels were initially proposed as primary pressure sensors, there is little experimental support for this contention. For example, although Piezo1 channels are present in vascular smooth muscle cells, they do not contribute to normal myogenic tone (Retailleau et al., 2015). Instead, there is now compelling evidence supporting a key role for G‐protein coupled receptors (GPCRs) in mechanosensation (Mederos y Schnitzler et al., 2008; Zou et al., 2004) and as vascular smooth muscle mechanosensors (Gonzales et al., 2014; Hong et al., 2016; Mederos y Schnitzler et al., 2008). Notably, angiotensin receptors (AT_1a_R and AT_1b_R) contribute to the generation of myogenic tone in rodent skeletal muscle, cerebral and mesenteric arteries (Hong et al., 2016; Pires et al., 2017), albeit whether these findings extend to human arteries remains to be determined. Further, many other GPCRs coupled to Gq/11, including the histamine receptor (H_1_R), muscarinic receptor (M_5_R), endothelin receptor (ET_A_R) and the vasopressin receptor (V_1A_R), all support mechano‐activation of TRP channels (Mederos y Schnitzler et al., 2008). These Gq/11 GPCRs use phospholipase C (PLC) signalling (Matsumoto et al., 1995; Osol et al., 1993) to activate vascular smooth muscle cell (VSMC) transduction channels and membrane depolarization (see Figure 1). Putative transduction channels include members of the transient receptor potential (TRP) family, TRPM4, TRPV1 and TRPP1 (Bulley et al., 2018; Earley et al., 2004; Phan et al., 2022; Reading & Brayden, 2007; Welsh et al., 2002), and the calcium‐activated chloride channel anoctamin‐1 (ANO1) (Bulley et al., 2012).

**FIGURE 1:**
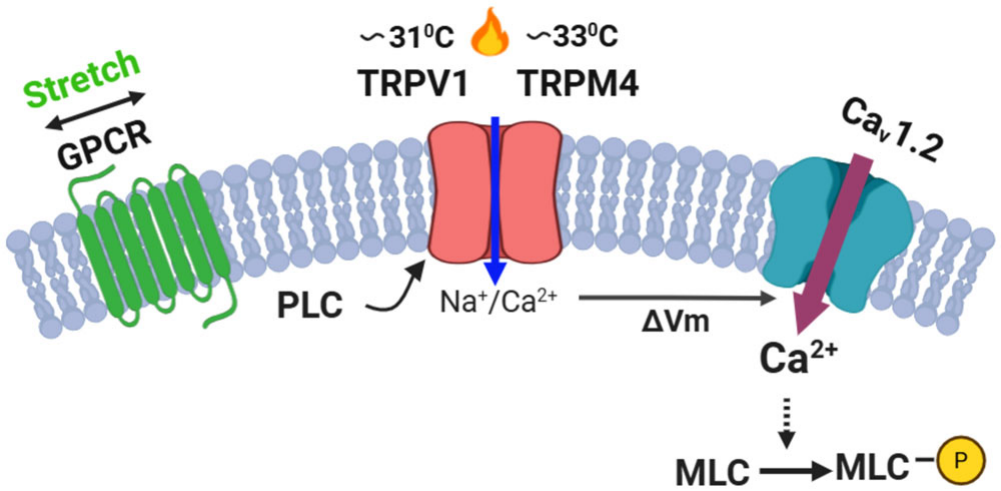
A model of thermo‐tone in skeletal muscle arteries. A putative signaling pathway for thermo‐tone in vascular smooth muscle. Mechanosensitive G‐protein coupled receptors (GPCRs) sense intraluminal pressure or stretch. Stretch‐induced PLC signalling (including protein kinase C‐mediated phosphorylation) sensitizes the thermo‐sensitive ion channels TRPV1 and TRPM4, tuning their temperature sensitivity to ∼31°C and 33°C, respectively. In turn, thermal activation of these ion channels triggers membrane depolarization (TRPV1 admits Na^+^/Ca^2+^ ions; TRPM4 admits only Na^+^ ions), activating voltage‐gated Ca^2+^ channels (Ca_V_1.2), raising myoplasmic Ca^2+^, and leading to the phosphorylation of myosin light chain necessary for contraction. Image constructed using Biorender.

## 2 MYOGENIC TONE IS STRICTLY THERMOSENSITIVE

Several of the ion channels implicated in myogenic tone, including TRPM4, TRPV1 and ANO‐1, are characteristically temperature sensitive (i.e., directly activated and sensitized by heat), raising the possibility that these channels confer an element of thermo‐sensitivity to myogenic tone. Indeed, Phan et al. (2025) found that myogenic tone in pressurized skeletal muscle arteries only developed when temperature was raised above 28°C. At higher temperatures, tone steeply increased reaching a maximum at ∼40°C. Analysis of myogenic tone in arteries isolated from gut, brain and skin similarly revealed a strict temperature dependence, and a steep rate of thermal activation. These findings are consistent with the results of earlier studies showing that myogenic tone fails to develop when pressurized arteries are studied at room temperature rather than at 37°C.

Interestingly, Phan et al. (2025) found that the half‐maximal response to temperature (*T*_1/2_) was tissue dependent; gut and brain arteries had a *T*_1/2_ of ∼35°C, skeletal muscle had a *T*_1/2_ of ∼33–34°C, hairy skin had a *T*_1/2_ of ∼31°, while glabrous skin had bi‐modal response with *T*_1/2_ of ∼24°C and ∼38°C. Since these values are close to the resting tissue temperatures for these respective arteries, Phan et al. suggested that tone is tuned to the local tissue temperature. But do vascular smooth muscle cells of peripheral arteries sense the tissue temperature or the core temperature (∼36°C–37°C) of inflowing blood? Indeed, there is a common misconception that arteries share a common blood temperature. However, due to heat loss and counter‐current heat exchange with veins, the temperature of the blood in small arteries (50–150 µm) of the skin and skeletal muscle approximates the temperature of the surrounding tissues (Bazett & Love, 1948; He et al., 2003). Of course, a very large increase in blood flow may dramatically warm peripheral tissues. This is indeed the case for glabrous skin, which covers heat‐exchange organs (e.g., the human hand or mouse paw). At cool ambient temperatures, the microcirculation of glabrous skin is under high sympathetic tone with minimal blood flow and a skin temperature just above the ambient level. During thermal stress, sympathetic tone is relieved and arterio‐venous anastomoses open resulting in a very large increase in blood flow (>500%), to radiate heat into the environment (Simmons et al., 2011). Due to this large hyperaemia, the glabrous skin rapidly approaches ‘core’ temperature (note that in the case of high thermal stress that produces sweating, the evaporative heat loss minimizes skin temperature changes). Thus, glabrous skin commonly exists in two distinct temperature states (Simmons et al., 2011) and the biphasic temperature sensitivity of myogenic tone appears to reflect these two states.

The tuning of tone to tissue temperature has important, haemodynamic implications. Since the *T*_1/2_ for tone resides in the steepest part of the temperature–tone relationship, myogenic tone will be maximally sensitive to even small thermal fluctuations.

## 3 WHAT ARE THE MOLECULAR UNDERPINNINGS OF THERMO‐TONE?

Temperature affects all proteins and enzymatic reactions; typically, a 10°C increase in temperature doubles the reaction rate with a temperature coefficient (*Q*_10_) of ∼2. In contrast, Phan et al. (2025) found that the *Q*_10_ for myogenic tone in arteries was ∼11–20, with skin arteries possessing a particularly high *Q*_10_. These results suggest a highly temperature‐sensitive mechanism consistent with a role for a specialized heat‐sensitive protein(s). Thermo‐TRP channels represent potential candidates, but temperature sensitivity may also reside in upstream GPCR mechanosensors and/or downstream L‐type Ca^2+^ channels and the contractile apparatus (Figure 1). Notably, Phan et al. (2025) found that the half‐maximal pressure for myogenic tone (*P*_1/2_) was not strongly affected by temperature (*Q*_10_ < 2). Similarly, the *T*_1/2_ for myogenic tone (∼34°C) was not affected by changing the intraluminal pressure between 40 and 100 mmHg. These results suggest that pressure and temperature are largely sensed independently. To confirm this, Phan et al. measured stretch‐induced intracellular Ca^2+^ release (a proximal event downstream of GPCR activation) in isolated vascular smooth muscle cells. Increasing the temperature, from 32°C to 37°C, did not significantly alter stretch‐evoked Ca^2+^ mobilization. Thus, mechanosensing in arteries is not highly temperature sensitive.

To assess the role of temperature on Ca^2+^ entry and contraction, Phan et al. (2025) measured depolarization‐induced artery constriction. Potassium chloride evoked near equivalent responses at 32°C and 37°C. These results suggest minimal thermosensitivity for these processes and are consistent with earlier studies of L‐type channel gating (Wang et al., 1991) and vascular smooth muscle contraction (Klemt & Peiper, 1978). Taken together, these data suggest that the locus for thermo‐tone likely resides with the transduction channels. Indeed, using sharp electrode impalement of skeletal muscle VSMCs, Phan et al. (2025) found that pressure‐induced membrane depolarization was temperature dependent; there was no pressure‐induced depolarization at 28°C but a marked response at 34°C.

Previous studies showed that TRPV1 and TRPM4 channels mediate most of the myogenic tone in skeletal muscle arteries, each contributing equally to the overall response (Phan et al., 2022). Using verified pharmacological antagonists (i.e., drugs that have no effect on arteries isolated from TRPV1 or TRPM4 knock‐out mice) Phan et al. (2025) showed that TRPV1 mediated thermo‐tone with a *T*_1/2_ of ∼31°C, while the TRPM4 component occurred at a *T*_1/2_ of ∼33°C. Thus, the overall tone in skeletal muscle arteries is the product of these discretely tuned TRP channels (Figure 1). In gut and brain arteries, TRPM4 was similarly tuned to ∼33°C. These central arteries do not express TRPV1 and therefore other thermo‐transduction channels, tuned to a slightly higher temperature, must contribute to explain the overall *T*_1/2_ (∼35°C) in these vessels. Potential candidates include ANO‐1 (Bulley et al., 2012) and TRPV2 (McGahon et al., 2016).

To verify thermo‐tone in vivo, Phan et al. (2025) turned to systemic blood pressure recordings in mice. In a previous study it was shown that intravenous administration of the TRPV1 antagonist, BCTC, acutely decreases blood pressure due to inhibition of the TRPV1‐mediated component of myogenic tone (Phan et al., 2022). Notably, Phan et al. (2025) found that this depressor effect of N‐(4‐tert‐butylphenyl)‐4‐(3‐chloropyridin‐2‐yl)piperazine‐1‐carboxamide (BCTC) was dependent on body temperature. At a normal core temperature of 36°C (skeletal muscle, 33°C), BCTC decreased blood pressure (BP) by ∼15 mmHg. However, when core temperature was lowered to 32°C (skeletal muscle, 29°C), BCTC failed to evoke a BP response, consistent with the absence of TRPV1‐mediated thermo‐tone.

Temperature activation of TRPV1 channels normally occurs above 40°C. Therefore, it may seem surprising that TRPV1 activation in VSMCs occurs with a threshold of ∼30°C. However, it is well known that phospholipase C signalling can lower the temperature activation threshold of TRPV1. Indeed, Phan et al. (2025) found that inhibiting PLC in skeletal muscle arteries, blocked most of the myogenic tone except for a residual fraction (15%) observed at very high temperatures (*T*_1/2_ above ∼39°C). TRPV1 and TRPM4 antagonists inhibited this residual tone. These findings suggest that in the absence of other stimuli, TRP channels are normally activated >39°C and that PLC signalling (activated by intraluminal pressure) reduces their temperature activation threshold.

Nonetheless, it remains somewhat puzzling that the *T*_1/2_ was invariant of intraluminal pressure (40‐100 mmHg). In the case of TRPV1 channel gating, agonists typically increase the sensitivity for temperature activation via allosteric mechanisms. Therefore, with higher pressures and greater PLC signalling one would expect a graded decrease in the *T*_1/2_. Phan et al. (2025) offer potential explanations. First, arterial thermosensitivity may be fixed because the TRP channels are already maximally sensitized to temperature at baseline pressures of ∼20–30 mmHg (perhaps by complete protein kinase C phosphorylation). Second, pressure/PLC signalling may generate agonists that activate TRP channels without altering their temperature sensitivity. Indeed, there is precedence for this as the lipid mediators diacylglycerol and lysophosphatidic acid both activate TRPV1 without modifying thermal sensing (Cao et al., 2013; Woo et al., 2008). Further studies are needed to determine the precise mechanism(s).

## 4 THERMOTONE COUNTERACTS THE EFFECTS OF TEMPERATURE ON BLOOD FLOW

Temperature prominently affects blood flow. Hyperthermia is well known to redirect blood from visceral organs to skin. An increase in core body temperature leads to sympathetic nerve‐mediated vasoconstriction of kidney and splanchnic vessels and vasodilation of cutaneous tissues (Costa et al., 2025; Rowell, 1974). Heating the skin also elicits a localized nerve mediated vasodilation. In addition, to these neurogenic mechanisms, heating directly affects blood flow. Indeed, the results of numerous studies performed over the past 70 years show pronounced effects of local heating on blood flow/vascular conductance in various tissues including skeletal muscle, bone, stomach and lung (Akyürekli et al., 1997; Barcroft & Edholm, 1943; Binzoni et al., 2012; Galletti et al., 1958; Heinonen et al., 2011; Koch Esteves et al., 2021; Salmon et al., 1959). For example, in dogs, intragastric heating in the range 15–38°C increased gastric blood flow ∼3‐fold (Salmon et al., 1959). This starkly contrasts with the well‐known haemodynamic response (sympathetic‐mediated vasoconstriction) in the splanchnic circulation to whole‐body heating. Similarly, local heating of dog lungs from 15°C to 37°C increased vascular conductance ∼2.4‐fold (Galletti et al., 1958). In humans, passive heating of limbs (in the range ∼25–37°C) increased skeletal muscle blood flow several‐fold (Akyürekli et al., 1997; Binzoni et al., 2012; Heinonen et al., 2011).

The underlying mechanisms for these effects are unclear but at least two theories have emerged. First, local heating may lead to arterial vasodilation. Potentially this could reflect enhanced nitric oxide production by vascular endothelium due to increased shear‐mediated signalling and/or heat‐induced release of ATP release from red blood cells (RBCs) (Kalsi & González‐Alonso, 2012). Second, local heating may increase blood flow by increasing blood fluidity (including RBC deformability and dispersion) (Rand et al., 1964; Shiga et al., 1979; Snyder, 1971; Williamson et al., 1975). Many studies report an approximately 3–4% increase in blood fluidity per °C. However, other studies have reported substantially larger effects (several‐fold per 10°C), particularly at high shear stresses (>6 dynes/cm^2^) (Williamson et al., 1975). Because shear stress in the smallest arterioles commonly exceeds 50 dynes/cm^2^, temperature‐dependent changes in blood rheology may be especially relevant in the microcirculation. Notably, the effects of temperature on blood fluidity appear to be more pronounced in situ than in vitro, where blood is typically analysed in glass capillaries. This difference may reflect interactions between RBCs and the endothelial surface layer (Pries & Secomb, 2005). Consistent with this idea, a 15% reduction in RBC deformability increases whole‐limb vascular resistance by approximately 75% in isolated, perfused rat hind limbs (Baskurt et al., 2004). Likewise, passive heating of human limbs over a physiological temperature range (∼25–37°C) increases skeletal muscle blood flow several‐fold (Akyürekli et al., 1997; Binzoni et al., 2012; Heinonen et al., 2011). The importance of temperature and blood rheology is further illustrated in sickle cell disease, where reduced RBC deformability impairs capillary transit especially in bones and joints (Jang et al., 2021) and cold exposure can precipitate vaso‐occlusive crises (Resar & Oski, 1991). Together, these findings suggest that tissue heating or cooling can substantially alter the frictional properties of blood and, consequently, microvascular conductance.

In summary, previous studies implicated a role for vasodilation, and/or fluidity in heat induced hyperaemia. To better understand the haemodynamic effects of local heating, Phan et al. (2025) performed intravital imaging in mice to directly measure skeletal muscle arterial diameter, and haemodynamic changes. Blood flow was measured using laser speckle contrast imaging, a technique which quantifies blood velocity based on the speckle pattern or blurring of incident infrared light reflected off RBCs. Notably, local heating from 28°C to 40°C decreased the artery diameter by up to 40%. Similar results were obtained with or without α1‐adrenergic blockade ruling out a contribution of sympathetic nerves, and thus the vasoconstriction is consistent with thermo‐tone. Surprisingly despite the vasoconstriction, Phan et al. (2025) recorded a large increase (∼2.5 fold) in blood flow through the heated arteries and veins. Analysis of the temperature dependence showed that the increase in blood flow was relatively small over the range 32°C to 38°C, but at higher temperatures, there was a surge in perfusion. Further, they repeated these experiments with myogenic tone blocked, using a calcium free medium combined with sodium nitroprusside. Under these conditions, with fully dilated arteries, heating still increased the blood flow by ∼2.5‐fold, peaking at 36°C.

These findings demonstrate several important features of arterial thermosensitivity, at least in the context of skeletal muscle. First, similar to experiments conducted in vitro, arteries in vivo constrict to thermal stimulation. Second, the observation that heat‐induced hyperaemia persists in fully dilated arteries appears to rule out vasodilation as an underlying mechanism. Instead, this finding supports temperature affecting the physical–chemical properties of blood including fluidity and endothelial surface interactions. Third, thermo‐tone controls blood flow over a narrow temperature range (30–39°C for skeletal muscle). At temperatures outside this range there is either no tone or tone has reached a maximum, and therefore, thermal hyperaemia is no longer restrained. This temperature range is tissue specific since thermo‐sensitivity is tuned to the resting tissue temperature of the resident arteries. Notably, arteries in skin, which undergoes dramatic temperature changes, have the steepest temperature activation. However, all tissues exhibit dynamic, regional temperature changes (outside of exercise) due to variations in metabolism; for example, brain temperature fluctuations (Kiyatkin, 2019). Moreover, core body temperature exhibits daily deviations up to 4°C from the thermal set point (Kiyatkin, 2019). Thus, thermo‐tone may function as a homeostatic mechanism to counteract the effects of changing temperature on local tissue blood flow. Consistent with this hypothesis, elevations in environment temperature are reported to increase the incidence of peripheral oedema (e.g. ankle swelling) in adults (Liu et al., 2016; Santa Cruz et al., 2023) and these symptoms are exacerbated in patients taking calcium channel blockers, which inhibit myogenic tone (Liang et al., 2022; Messerli, 2001).

## 5 SUMMARY AND FUTURE PERSPECTIVES

Myogenic tone is a vital autoregulatory mechanism for controlling tissue blood flow. Recent work by Phan et al. (2025) demonstrated that the myogenic tone of resistance arteries is intrinsically thermosensitive and tuned to the local tissue temperature. This effect is mediated by vascular smooth muscle thermosensitive TRP channels, including TRPV1 and TRPM4, and exhibits a steep temperature dependence (*Q*_10_ ≈ 10–20). The myogenic response, per original description, stabilizes blood flow over a discrete range of intraluminal pressures. The work by Phan et al. reveals that myogenic tone also stabilizes blood flow over a discrete range of temperatures. Although pressure and temperature can be modelled separately, both are required for myogenic tone. These stimuli are sensed independently, and the signals integrated by the transduction channels to determine the extent of vascular tone. These findings have intriguing implications for local/regional blood flow regulation and our understanding of thermal physiology. Several, outstanding questions remain that may be addressed in future studies. These include the identity of the arterial thermosensitive channels in different tissues, the precise mechanism of the temperature tuning, and whether altered thermo‐tone contributes to dysregulation of local blood flow in disease states.

## AUTHOR CONTRIBUTIONS

Thieu X. Phan and Gerard P. Ahern were responsible for the concept, drafting and revising of the manuscript. All authors have read and approved the final version of this manuscript and agree to be accountable for all aspects of the work in ensuring that questions related to the accuracy or integrity of any part of the work are appropriately investigated and resolved. All persons designated as authors qualify for authorship, and all those who qualify for authorship are listed.

## CONFLICT OF INTEREST

The authors declare no conflicts of interest.

## GENERATIVE AI STATEMENT

No generative AI was used in the drafting/editing of the manuscript.
